# The flexibility of the transverse arch of the forefoot on the forefoot loading in the flat feet deformity

**DOI:** 10.1186/1757-1146-7-S1-A48

**Published:** 2014-04-08

**Authors:** Shintarou Kudo, Yasuhiko Hatanaka

**Affiliations:** 1 Graduate school of medical science, Suzuka University of Medical Science, Suzuka, Mie, 510-0293, Japan; 2 Department of Physiotherapy, Suzuka University of Medical Science, Suzuka, Mie, 510-0293, Japan

## Background

For the flat foot deformity, the custom made foot orthosis supported medial longitudinal arch using the navicular pad. And we experienced many cases that addition the metatarsal pad which supported transverse arch of the forefoot was more effective treatments. However, the flexibility of the transverse arch in the flat foot deformity was unclear. The aim of this study was to clarify the flexibility of the transverse arch of the forefoot in the flat foot deformity on the forefoot loading condition.

## Methods

The sixty-one feet of fifty-two normal volunteers (32 males and 19 females, 22.0±3.8 years old) were participated in this study. They were categorized normal feet group and flat feet group by their foot posture and medical history around the foot. The ten spherical 4mm diameter skin markers were mounted over the each metatarsal heads and bases. Measurement foot stance forward and body weight lorded on the forefoot as well as possible with whole plantar surface in contacted with the floor. Foot motion was recorded using four Hi-definition digital video cameras with 60 Hz. The ground reaction force and plantar foot distributions were recorded using the force plate (Anima.co, Japan) and the Win-pod (Medicaputures s.a.s. France) with 180 Hz, respectively. The each marker was manually digitized using the Flame Dias4. The calibration flame was used 64mm^3^ acryl cubes. The distance from the first to the fifth metatarsal head and base were calculated as forefoot and mid foot width (FFW and MFW), respectively. The transverse arch height of both the metatarsal head and base (TAHH and TAHB) was defined as the distance from the second metatarsal head and base to the floor divided by FFW and MFW, respectively. The difference FFW and TAHH, MFW, TAHB between rearfoot and forefoot loading were measured as Diff- FFW and Diff-TAHH, Diff-MFW, Diff-TAHB. The four parameters were assessed among the two groups using Mann-Whitney test.

## Results

The Diff-FFW and the Diff-TAHH had significant difference between two groups. And the Diff-MFW and the Diff-TAHB didn’t have significant difference (Table 1).

**Table 1:** The difference four parameters between standing and forefoot loading [median (25%-75%)].

	Normal feet	Flat feet	p-values
Diff-FFW	1.52 % (1.17-2.11)	2.08 % (1.78-3.07)	p<0.001

Diff-TAHH	1.79 % (1.28-2.45)	2.78 % (2.15-3.63)	p<0.001

Diff-MFW	1.31 % (0.94-1.66)	1.17 % (0.94-1.66)	p=0.21

Diff-TAHB	3.20 % (2.33-4.68)	3.18 % (2.68-4.23)	p=0.46

## Conclusion

It was needed for physical therapy of the foot to understand kinematics of the forefoot in the dynamic condition. The forefoot flexibility with the flat foot deformity was greater than that with the normal feet. It might be better to insert the metatarsal pad which aimed to increase the rigidity of the forefoot.

